# Immediate Esthetic Build-Up (IEB) for Anterior and Premolar Teeth During Root Canal Therapy

**DOI:** 10.7759/cureus.93611

**Published:** 2025-09-30

**Authors:** Abdulkarim A Temsah

**Affiliations:** 1 Dental Department, Specialized Medical Center Hospitals, Riyadh, SAU

**Keywords:** coronal seal, endodontics, esthetics, immediate esthetic build-up, pretreatment buildup, provisional restoration, root canal therapy, urgent endodontics

## Abstract

Immediate root canal therapy on anterior and premolar teeth often leads to esthetic compromise, particularly when multi-visit treatment is needed or coronal structure is significantly damaged. Existing provisional approaches, such as silicone indices, are often impractical in urgent settings due to time constraints. The immediate esthetic build-up (IEB) technique uses a glycerin-coated gutta-percha to serve as a canal projector, defining the access cavity during provisional restoration. Composite or glass ionomer is applied around the cone, with the buccal surface always restored using composite. After the restorative material has set, the cone is removed, leaving a precise access cavity. The IEB serves two purposes: (1) as a pretreatment buildup for structurally compromised teeth to enable secure rubber dam placement and (2) as an esthetic inter-appointment seal during multi-visit therapy to maintain canal patency and coronal integrity. The IEB technique provides a simple, cost-effective method to restore esthetics, facilitate isolation, and preserve tooth structure during urgent endodontic treatment. This practical approach, relying on readily available dental materials, offers predictable outcomes and can be easily integrated into the routine practice of general dentists managing anterior and premolar cases in both pretreatment and multi-visit scenarios.

## Introduction

Current endodontic literature emphasizes the critical need for provisional restorations in immediate cases, particularly in the esthetically critical anterior region, to protect function, maintain appearance, and enable efficient workflow. Despite this, conventional provisional restorations documented in the literature, often fabricated under urgent conditions, frequently lack the requisite durability, marginal seal, or satisfactory esthetics [1].

The standard approach using a silicone index requires additional chair time and results in a core that must later be blindly drilled through, risking iatrogenic removal of sound tooth structure or compromise to the final restoration. Simpler temporary filling plugs (e.g., glass ionomers or Cavit) provide limited coronal seal, compromise esthetics, and necessitate further restorative steps later in the treatment sequence.

The immediate esthetic build-up (IEB) technique, a refined protocol based on a preliminary concept introduced in 2012 [2], directly addresses these clinical shortcomings. It is a simplified, reliable, and cost-effective approach for two essential scenarios: pre-treatment buildup for severely compromised teeth and immediate inter-appointment sealing in esthetically sensitive areas.

The objective of this technical report is to detail the precise, step-by-step clinical protocol for the IEB technique, demonstrating its application, material usage, and unique advantages in providing both a durable, esthetic provisional restoration and a predictable access pathway for multi-visit root canal therapy.

This article is an expanded report of a poster presentation titled "Alternative Way of Maintaining Esthetics During Root Canal Treatment" at the 19th International Dental Congress in May 2012.

## Technical report

The IEB technique is applied in two distinct clinical scenarios: as a pretreatment buildup in teeth with compromised coronal structure to facilitate rubber dam placement, and as an inter-appointment seal in multi-visit cases to maintain esthetics and coronal integrity. The following numbered steps outline the procedure, with corresponding figures.

Step 1 - Initial Preparation and Evaluation

**Figure 1:**
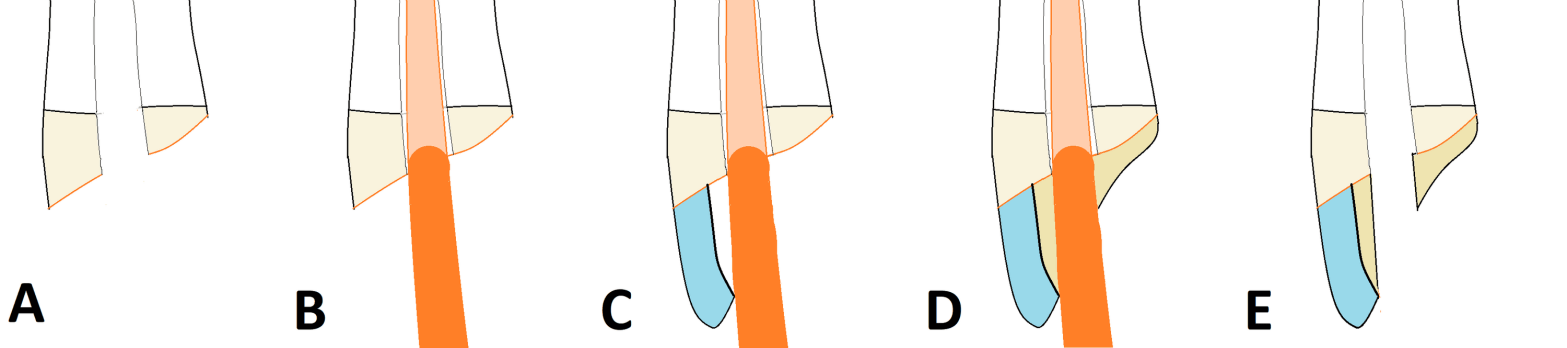
The immediate esthetic build-up (IEB) technique. The various stages of the IEB procedure are shown, from initial preparation to final access cavity formation. (A) Initial Preparation: Upper central incisor with adequate remaining coronal structure is prepared. Any existing carious tissue or compromised restoration is completely removed. (B) Canal Projection: A precisely sized, glycerin-coated gutta-percha cone is placed passively into the canal orifice to serve as a non-adhesive access projector, ensuring the tip is seated below the level of the future coronal seal. (C) Coronal Build-Up: Flowable and/or packable composite material is applied incrementally to the buccal aspect, fully stabilizing the cone and initiating the restoration of the tooth's anatomical contour. (D) Completed Build-Up: The restorative material is cured and fully polished, completely encasing the cone. The resulting provisional restoration provides excellent esthetics, marginal seal, and function. (E) Access Cavity Formation: The gutta-percha cone is removed using an excavator or gentle instrument, revealing a precise, pre-defined access cavity that is fully protected by the durable composite margin. The access is now ready for subsequent endodontic treatment. The figure is a unique creation by the author for this report.

After removing all carious tissue and unsupported enamel, the tooth is evaluated for restorability. After future crown preparation, there must be at least 1 mm of dentin thickness and a minimum of 1.5 mm vertical height of coronal structure to provide an adequate ferrule [3,4] (Figure 1A).

Step 2 - Placement of Gutta-Percha

A large segment of a gutta-percha cone (Meta Biomed) is selected to serve as a predictable root canal projector. In teeth with compromised coronal structure, the cone is inserted before rubber dam placement. For multi-visit cases, a master tapered gutta-percha cone is placed into the canal after initial endodontic access, cleaning, and shaping. A glycerin-based separating medium (Liquid Strip, Ivoclar Vivadent) is applied to the coronal tip of the cone before insertion to act as a release agent (Figure 1B).

Step 3 - Coronal Build-up

An esthetic provisional restorative material is used to build up the coronal tooth structure around the inserted gutta-percha cone. The buccal aspect is always built up with a composite resin (Filtek Z250, 3M) (Figure 1C), while the remaining structure is filled with either flowable composite (Filtek Flow, 3M) or glass ionomer cement (GIC; Ketac Fil, 3M) (Figure 1D). When using resin-based materials, a bonding agent (Scotchbond Universal, 3M, St. Paul, MN) is applied beforehand.

Step 4 - Access Cavity Creation and Isolation

Once the restorative material has fully set, the lubricated gutta-percha cone is removed, leaving a round, pre-defined access cavity within the provisional restoration. For the pretreatment buildup scenario, this allows secure placement of a rubber dam for final isolation and continued endodontic therapy (Figure 1E).

Step 5 - Provisional Seal

Between appointments, the access cavity can be sealed with a reliable temporary filling material such as Cavit (3M) to ensure coronal integrity.

Note: Equivalent materials from other manufacturers may be substituted in all steps.

## Discussion

The clinical significance of the IEB protocol centers on its ability to concurrently support two essential pretreatment goals: preventing contamination and ensuring a secure coronal seal. Unlike conventional provisional methods, which often lack the durability, marginal seal, and satisfactory esthetics required in these time-sensitive cases [5,6], the IEB technique directly addresses this deficiency. A durable, esthetic provisional restoration protects the endodontic space from bacterial ingress and irrigant percolation. Recent research indicates that root canal irrigants, when used appropriately, do not affect the adhesive bond strength of pre-endodontic resin restorations [7].

When coronal tooth structure is severely compromised, the IEB provides a stable platform for early rubber dam placement, greatly improving isolation compared to traditional methods. Alternative isolation strategies, such as the split rubber dam technique, can be helpful but often fail to achieve a fully aseptic, moisture-free field [8].

Incorporating a glycerin-coated gutta-percha cone within this build-up creates a predictable, precise access opening for subsequent appointments. This eliminates blind drilling through bulky provisional fillings - reducing unnecessary removal of sound tooth structure, avoiding iatrogenic damage, and preserving pulp chamber anatomy. The pre-defined access maintains a continuous, tapered shape that facilitates insertion of paper points and gutta-percha. The built-up structure itself can be used to facilitate a future post placement without being removed, saving valuable chairside time.

The IEB is a practical, cost-effective alternative to specialized systems, such as 3D-guided endodontic projectors, devices using custom guides, or the Projector Endodontic Instrument Guidance System (PEIGS), which offer precision but are often impractical due to cost and limited availability [9,10]. It is also an alternative to other techniques, such as those that use hypodermic needles as sleeves, that may create an orifice lacking in taper [11]. A key advantage of the IEB is its focus on immediate esthetic build-up, a benefit often not emphasized in reports of similar canal projection techniques for posterior teeth.

Beyond its role in canal projection, the glycerin coating has an additional polymerization advantage. Glycerin’s water solubility allows it to be rinsed away easily, and when applied during curing, it minimizes the oxygen inhibition layer on the composite surface adjacent to the cone. This results in improved surface monomer conversion and enhanced hardness, as demonstrated in prior research [12].

This technique further contributes to the long-term prognosis of the treated tooth by preserving maximum tooth structure and reducing fracture risk. It also eliminates the need for a time-consuming silicone key, utilizing commonly available dental materials. Equivalent alternative brands can be used, making the approach broadly applicable and adaptable to different clinical settings.

Table 1 outlines a detailed comparison of the IEB technique against conventional provisional methods (e.g., silicone index, temporary filling plugs) and more specialized canal projection methods (e.g., PEIGS, hypodermic needles), highlighting the unique advantages of IEB in terms of cost, esthetics, and access predictability.

**Table 1:** Comparative summary of immediate esthetic build-up (IEB) vs. alternative methods. This table details the relative advantages and limitations of the IEB technique compared to conventional provisional restorations (silicone index, temporary filling plugs) and specialized canal projection methods (PEIGS, hypodermic needles), highlighting differences in cost, esthetics, and access predictability.

Feature	Immediate Esthetic Build-Up (IEB) Technique	Silicone Index Technique (Provisional Core Build-up)	Temporary Filling Plugs (e.g., Cavit, GIC)	PEIGS (Projector Endodontic Instrument Guidance System)	Hypodermic Needles as Sleeves Technique
Esthetics & Form	Excellent. Restores full anatomical and esthetic contour immediately; uses high-quality restorative composite.	Fair/Good. Requires separate time for fabrication; contour depends on the quality of the index and material used.	Poor. Relies on generic temporary materials; only provides a plug form, lacking anatomical contour.	None. Pure access guidance system; requires a separate restoration for esthetics/seal.	None. Pure access guidance system; requires a separate restoration for esthetics/seal.
Access Predictability	Excellent (Low-Tech). Glycerin-coated gutta-percha creates a defined, pre-shaped, tapered access cavity.	Poor. Requires blind drilling through the core material, risking perforation or unnecessary tooth structure removal.	Poor. Requires blind drilling through bulk plug material, risking perforation or unnecessary tooth structure removal.	Excellent (High-Tech). Highly predictable, often 3D-printed and digitally guided for minimal structure removal.	Good (Low-Tech). Provides a basic, rigid tunnel, but may lack optimal taper or size control.
Time & Materials	Low Cost/Low Complexity. Uses readily available clinical materials (gutta-percha, composite, glycerin).	Variable/Moderate Complexity. Requires extra steps (silicone putty, custom tray) and chair time for index fabrication.	Low Cost/Low Complexity. Uses basic plug materials (Cavit/GIC).	High Cost/High Complexity. Requires specialized software, planning, and fabrication (often 3D printing).	Low Cost/Low Complexity. Uses readily available materials (needle/syringe) but is technique-sensitive.
Key Limitation	Technical Sensitivity. Sensitive to moisture, operator technique, and anatomical constraints (calcified canals, oval shape risk).	Time/Complexity. Time-consuming fabrication process; outcome depends on index quality.	Poor Esthetics. They inherently lack the esthetic properties required for anterior restorations.	Cost & Accessibility. Impractical for routine use; requires significant capital investment and specialized training.	Limited Taper/Size. Creates non-tapered or wide orifices; risk of iatrogenic removal of sound tooth structure when removing the sleeve.

Despite its numerous advantages, the IEB technique is not without limitations. The reliance on a glycerin-coated gutta-percha cone means the procedure is contraindicated in cases of calcified root canals or when a significant obstruction exists at the canal orifice, as the cone must be seated passively and securely to accurately define the access cavity. Furthermore, in premolar teeth with two separate canals (e.g., buccal and lingual/palatal), the technique's application would require adjustment of the orifices to connect them as a single unified access cavity to enable better cleaning and shaping of any intervening isthmus. Like all adhesive restoration techniques, the method is sensitive to moisture and operator technique, requiring meticulous application of the bonding agent and composite materials. Specifically, extreme caution is necessary during the application of the bonding agent and any subsequent flowable restorative material to prevent accidental flow and polymerization within the canal walls, a risk that is significantly increased in canals with an oval cross-section due to the space around the round gutta-percha cone. The technique also relies on the technical precision of the glycerin application: if excessive glycerin residue is left in the defined access cavity, it may compromise the subsequent temporary coronal seal.

## Conclusions

This simple and cost-effective method provides an elegant solution for maintaining esthetics and preserving tooth structure in both pretreatment situations and during multi-visit endodontic therapy on compromised anterior and premolar teeth. By creating a predictable and esthetic provisional restoration, the IEB technique improves patient compliance, simplifies rubber dam isolation, and minimizes the risk of iatrogenic damage. Its reliance on readily available materials makes it a practical and accessible alternative to more complex or specialized methods, making it a valuable procedure for general practitioners to consider as a standard protocol in such cases. However, it is important to note that the technique is sensitive to operator technique and anatomical constraints, particularly in calcified or multi-canalled premolar teeth. Future randomized controlled trials are warranted to formally compare the long-term clinical and cost-effectiveness of the IEB technique against conventional provisional methods.
